# Application of adipose-derived stem cells in ischemic heart disease: theory, potency, and advantage

**DOI:** 10.3389/fcvm.2024.1324447

**Published:** 2024-01-19

**Authors:** Weizhang Xiao, Jiahai Shi

**Affiliations:** Department of Cardiothoracic Surgery, Affiliated Hospital and Medical School of Nantong University, Nantong, China

**Keywords:** adipose-derived mesenchymal stem cells, stem cell transplantation, ischemic heart disease, differentiation, exosomes, paracrine

## Abstract

Adipose-derived mesenchymal stem cells (ASCs) represent an innovative candidate to treat ischemic heart disease (IHD) due to their abundance, renewable sources, minor invasiveness to obtain, and no ethical limitations. Compared with other mesenchymal stem cells, ASCs have demonstrated great advantages, especially in the commercialization of stem cell-based therapy. Mechanistically, ASCs exert a cardioprotective effect not only through differentiation into functional cells but also via robust paracrine of various bioactive factors that promote angiogenesis and immunomodulation. Exosomes from ASCs also play an indispensable role in this process. However, due to the distinct biological functions of ASCs from different origins or donors with varing health statuses (such as aging, diabetes, or atherosclerosis), the heterogeneity of ASCs deserves more attention. This prompts scientists to select optimal donors for clinical applications. In addition, to overcome the primary obstacle of poor retention and low survival after transplantation, a variety of studies have been dedicated to the engineering of ASCs with biomaterials. Besides, clinical trials have confirmed the safety and efficacy of ASCs therapy in the context of heart failure or myocardial infarction. This article reviews the theory, efficacy, and advantages of ASCs-based therapy, the factors affecting ASCs function, heterogeneity, engineering strategies and clinical application of ASCs.

## Introduction

Ischemic heart disease (IHD), the most prevalent cardiovascular disease, is the culprit in majority of acute heart events, and remains the leading cause of death globally (1). This condition arises from the stenosis and blockage of coronary arteries, which inevitably leads to a decreased blood supply to the heart, resulting in irreversible damage to myocardium filling with necrotic cardiomyocytes. Significant decline in cardiac function develops, progresses to heart failure and death ultimately. Despite remarkable progress in drug development and advancements in interventional and surgical treatments over the centuries, neither of them can reverse the myocardial necrosis caused by extended periods of hypoxia. Given the limited regenerative capacity of cardiovascular tissue after injury in mammals, stem cells have emerged as a promising strategy for treating IHD.

Mesenchymal stem cells (MSCs) are a subset of stem cell family that reside in virtually all tissues with specific stem cell niches in the human body (2). MSCs can be obtained from various depots, including bone marrow (BM-MSCs), umbilical cord (UC-MSCs), adipose tissue. Among them, adipose-derived mesenchymal stem cells (ASCs) are particularly appealing due to their high accessibility, minimally invasive harvesting, high stem cell density, low immunogenicity, and no ethical restrictions (3). In this review, we highlight the advantages of ASCs-based therapy compared to other MSCs and explore the heterogeneity of ASCs. We focus on the therapeutic potential of ASCs in treating IHD through differentiation into functional cells, puissant paracrine that facilitates immunomodulation and angiogenesis, as well as engineering strategies and current clinical applications of ASCs.

## What are the advantages of ASCs compared with BM-MSCs and UC-MSCs?

Besides readily access and high yield, ASCs present some unique biological features compared to BM-MSCs and UC-MSCs. Table 1 summarizes the differences among these three types of MSCs. Specifically, to exclude the impact of donor health status, origin of MSCs, and culture strategy on the growth profile and senescence of MSCs, researchers isolated BMSCs and ASCs from the same donor. Their findings suggested that compared with BM-MSCs, ASCs displayed faster proliferation, shorter doubling time, and postponed senescence featured with longer telomere and lower expression of p16^ink4a^ (a characteristic gene of senescence) (4). Intriguingly, ASCs exhibited preferential adipogenesis, while BMSCs retained superior osteogenesis, which might be related to their distinct origin (5–9). Furthermore, ASCs have shown enhanced improvement in wound healing compared to their bone marrow-treated counterparts, suggesting a superior paracrine potential (10). In addition, higher concentration of cytokines including interlukin-6 (IL-6) and transforming growth factor-β (TGF-β) was observed in the supernatant of ASCs compared to BM-MSCs (11). ASCs also exerted potent immunosuppressive effects on T cells and DCs, along with upregulation of indoleamine 2,3-dioxygenase (IDO), a marker of MSCs immunosuppression on mononuclear cells (11–13), insinuating a stronger immunosuppressive capacity.

**Table 1:** Different characteristics of ASCs compared with BM-MSCs and UC-MSCs.

Features	ASCs 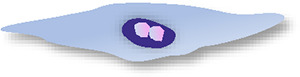	BM-MSCs 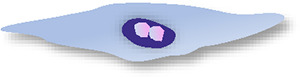	UC-MSCs 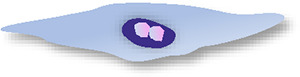
Availability	High	Low	Medium
Process of procurement	Safe	Invasive, painful, risky of infection	Safe, non-invasive
Cell yield	High	Low	High
Doubling time	Controversial		
Senescence rate	Controversial		
Adipogenesis	High	Medium	Low
Osteogenesis	Medium	High	Medium
Chondrogenesis	Controversial		
Pro-angiogenesis	High	Medium	Medium
Immunomodulation	High	Medium	Medium

Meanwhile, it is undeniable that the accessibility, high yield, and cultural expandability of cell candidates *in vitro* are crucial for successful cell-based therapy. In this respect, ASCs offer several advantages, including abundant sources, efficient scalability, low immunogenicity, and powerful immunosuppressive capacity. These features make ASCs suitable for both autologous and allogeneic transplantation and have exhibited exciting prospects for the commercialization of stem cell-based therapy.

## How does it work: the mechanism of ASCs treatment for IHD?

**Figure 1:**
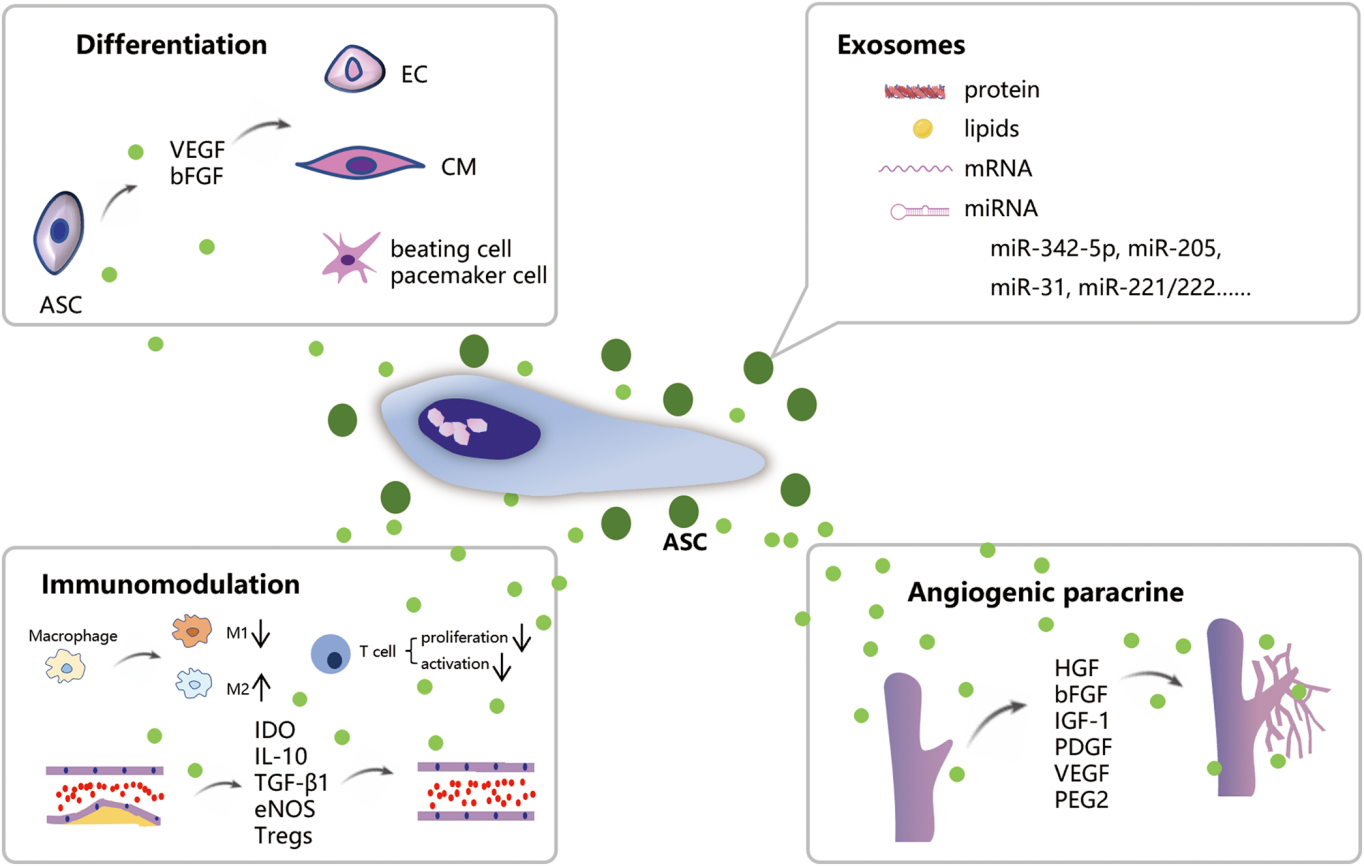
Schematic illustration of the mechanism of ASCs treatment for IHD. ASCs can differentiate into CMs, ECs, and beating cells/pacemaker cells. ASCs own robust paracrine function which promotes angiogenesis and immunomodulation on macrophage polarization and atherosclerosis. Exosomes released from ASCs contain biologically active substances such as proteins, lipids, and mRNA which possess comparable therapeutic effects on IHD.

The views on the mechanisms behind the use of ASCs in the treatment of IHD continues to evolve over time. These developing theories will undoubtedly drive the better administration of ASCs. As illustrated in Figure 1, ASCs possess competent differentiation and paracrine potential, enabling them to effectively treat IHD from multiple aspects.

## Is differentiation potential the primary mechanism behind ASCs-based therapy?

The differentiation of ASCs into other cell lines was once perceived as the key mechanism behind ASCs-based therapy. Beyond their fundamental trilineage potential *in vitro*, ASCs have been widely observed to differentiate into endothelial cells (ECs) and cardiomyocytes (CMs) under certain circumstances (14, 15). For instance, Kendra Clark, et al. observed the endothelial differentiation of ASCs when cultured in endothelial differentiation media. A specific 3D culture system enhanced this process, and a high concentration of vascular endothelial growth factor (VEGF) further augmented endothelial differentiation (16). Meanwhile, hypoxia treatment, which mimics the native physiological niche of ASCs, was found to facilitate the endothelial lineage differentiation of ASCs under stimulation with VEGF and bone morphogenetic protein-4 (BMP4) (17). Except VEGF, basic-fibroblast growth factor (bFGF) is another effective inducer of ECs differentiation from ASCs, with an induction rate exceeding 85% (18). Moreover, shear stress, which simulated the ECs environment *in vivo*, has been found to facilitate endothelial differentiation of ASCs and upregulate the expression of anti-thrombogenic markers (19). In animal models, plentiful human CD31-positive cells and regenerated blood vessels were observed in mouse hindlimbs injected with human stromal-vascular fraction (SVF), the origin of ASCs, suggesting that SVF cells have the potential to differentiate into endothelial cells and promote vascular regeneration directly (20). In terms of mechanism, miR-145 was identified as a key component in EC differentiation of ASCs, Upregulation of miR-145 suppressed the EC differentiation via regulating ETS1 expression, which can be reversed by overexpression of ETS1 (21). UTP is considered as another regulator of ASCs cardioprotective property in IHD, which not only enhances the revascularization in ischemic myocardium, but also directly promotes the endothelial differentiation of ASCs (22, 23).

On the other hand, although it seems more difficult for ASCs to differentiate into cardiomyocyte-like cells compared to endothelial cells, there are still numerous studies that have discovered this differentiation potential of ASCs. For instance, in 2015, HIROKI, et al. have found ASCs derived from cardiac adipose tissue could directly differentiate into cTnT-positive cells *in vivo*, while ASCs from subcutaneous, visceral, and subscapular adipose tissue failed to differentiate into cardiomyocytes (15). This finding indirectly highlights the challenges associated with cardiomyocyte differentiation of ASCs. To effectively induce ASC differentiation into cardiomyocytes, Zhang and his colleagues designed a type of gelatin/polycaprolactone fibers which promoted cardiomyocyte differentiation and facilitated ASCs proliferation as well (24). Wang, et al. discovered that chitosan can facilitate cardiac differentiation of ASCs through enhancing the collagen synthesis (25). They developed an injectable chitosan hydrogel as a deliverer for ASCs, which not only enhanced the cardiomyocyte differentiation, but also improve the survival of ASCs in infarcted hearts. Furthermore, Yan, et al, cultured ASCs on a polylactic acid (PLA) nanopillar array, then observed distinct cardiomyocyte-like cell markers and the induced ASCs injected into the myocardium exhibited significant protective effects on ischemic myocardium (26). Therefore, the differentiation of ASCs into cardiomyocytes is not only possible, but also feasible and holds great promise.

Besides, ASCs possess the potential of differentiating into pacemaker cells, giving insight into the treatment of arrhythmia, one common complication of IHD (27). To achieve that, scientists added certain differentiation factors such as BMP4 into the culture medium (28), or transfected ASCs with specific genes closely related to sinoatrial node function, such as TBXs (29, 30).

Nevertheless, the statement that ASCs differentiate into functional cells is not rigorous. It seems more appropriate to call these differentiated cells “endothelial-like cells, cardiomyocyte-like cells, and pacemaker-like cells”. Meanwhile, ASCs implanted into the ischemic myocardium, either locally or systemically, will struggle with a harsh microenvironment characterized by hypoxia, elevated oxidative stress, free radical production, limited nutrient supply, and the presence of proinflammatory cytokines, along with infiltration of immune cells (31). As a result, only a few ASCs are retained within proximity to the graft site and effectively integrate into the affected host tissue (32). Therefore, it may be the other mechanism rather than differentiation capability dominates the ASCs-based therapy.

## What is the role of proangiogenic paracrine of ASCs?

Since the implanted cells hardly survive in the tough soil *in vivo*, the potent paracrine of ASCs may be responsible for the compensatory angiogenesis in the ischemic area. Numerous studies have revealed that ASCs produced a variety of cytokines that promote angiogenesis, including VEGF, bFGF, hepatocyte growth factor (HGF), platelet-derived growth factor (PDGF), insulin-like growth factor-1 (IGF-1), et al. (33–36). Among these various angiogenic cytokines, VEGF is particularly notable due to its close relation to angiogenesis. In vitro studies have shown that ASCs generate high concentration of VEGF into the supernatant, especially when cocultured with human umbilical vein endothelial cells or endothelial progenitor cells (14, 37). Furthermore, under the hypoxic conditions which imitated the *in vivo* environment of IHD, ASCs secreted higher amounts of VEGF, HGF, and stromal-derived factor-1 (SDF-1) compared to normoxic conditions (38–40). The lesion-associated hypoxia, which curbed the survival of engrafted stem cells, would inevitably lead to the activation of hypoxia-inducible factor 1, resulting in increased VEGF release, its classical target gene (41). Additionally, pretreating ASCs with endothelial differentiation medium dramatically enhanced their proangiogenic action by increasing the amount of microvesicles released by ASCs (42). The underlying mechanism involves the transfer of microRNAs in microvesicles from ASCs to the vascular endothelial cells.

The potent proangiogenic function of ASCs has also been confirmed *in vivo*. In murine models of skin pressure ulcers and ischemic hindlimbs, injection of ASCs led to the formation of high-density capillary and branched tubelike structures, accompanied by accelerated recovery (43–45). Similarly, in a rat model of myocardial infarction (MI), administration of ASCs increased angiogenesis in the ischemic area, decreased infarct size, and improved heart function (46). Comparable results were observed in the swine model, which was closer to humans (47). Furthermore, a clinical study conducted in Japan utilized autologous ASCs for patients with limb ischemia. The application of ASCs significantly improved the clinical outcomes through angiogenesis without adverse events (48). Collectively, angiogenesis, driven by the robust paracrine action of ASCs, has been identified to play a primary role in the ASC-based IHD therapy.

## How does ASCs achieve therapeutic action via immunomodulation?

Following an MI, the innate immune response is triggered, characterized by the recruitment and infiltration of massive inflammatory immune cells, such as monocytes-derived-M1 macrophages, which eventually transformed into anti-inflammatory M2 macrophages, accompanied by the release of various pro- and anti-inflammatory cytokines. Studies have shown that coculture of ASCs and macrophages *in vitro* significantly induced macrophages toward reparative M2 phenotype and altered their cytokine secretion (49). Additionally, ASCs-based therapy has been found to increase the percentage of M2 macrophages in both spontaneously hypertensive rats and ischemic cardiomyopathy models, leading to improved disease prognoses (50, 51), indicating the therapeutic potential of ASCs in modulating the innate immune system.

Importantly, ASCs transplantation has been extensively proposed as an effective approach to treat atherosclerosis (ATH), which underlies many vascular disorders, such as aneurysm, atherosclerosis obliterans, and IHD. The protective potency of ASCs on ATH is primarily attributed to their robust paracrine action, which involves the release of various bioactive factors, such as IDO, TGF-β1, and IL-10, along with decreased release of pro-inflammatory cytokines, including TNF-α and IL-1β (52, 53). IDO is widely considered to suppress the proliferation of T cell and NK cells, impede TH17 differentiation and DCs maturation (13, 54), while IL-10 blocks macrophage activation, disrupts the production of pro-inflammatory cytokines and matrix metalloproteinase (MMP), and represses T cell proliferation, thereby impacting the local inflammatory response within the lesion (55). Moreover, TGF-β1 is involved in the decrease of NK cells proliferation and the MSC-mediated induction of CD4 ^+ ^CD25 ^+ ^Foxp3^+^ regulatory T cells (Tregs) (53). Tregs then exert a protective effect by suppressing the function of Th1/Th2 cells and DCs and promoting the stability of atherosclerotic lesions by inhibiting the expression of MMP-2 and MMP-9, which are crucial in degrading extracellular matrix proteins (56). Intriguingly, T cell activation was significantly inhibited when cocultured with ASCs under hypoxia, manifested by the upregulation of anti-inflammatory cytokines including PDCD1, Foxp3, and TGFβ1, and downregulation of genes involved in pro-inflammatory response such as IL2 and IFNG (57). In animal models of ATH, ASCs transplantation dramatically reduced the total cholesterol, triglyceride, and low-density lipoprotein cholesterol levels, while increasing high-density lipoprotein cholesterol levels, and ameliorating the pathological status of aortic ATH (58). Therefore, ASCs exert a positive effect on IHD from the immunopathology.

## ASC-derived exosomes and IHD

Exosomes are nano-vesicles secreted by cells into the extracellular environment, containing biologically active substances such as proteins, lipids, and mRNA. In recent years, ASC-derived exosomes have demonstrated similar therapeutic effects on IHD as ASCs. For instance, Xing, et, al. revealed ASCs-derived exosome delayed the development of ATH through a miR-342-5p mediated endothelial protection (59). In animal MI models, exosomes extracted from ASCs exerted therapeutic effect by promoting angiogenesis via miR-205 and miR-31 (60, 61), preventing cardiomyocyte apoptosis and hypertrophy via miR-221/222 (62–64), enhancing M2 macrophage polarization (65), ameliorating excessive ROS production, and attenuating cardiac fibrosis (66). To enhance the retention of exosomes in ischemic myocardium, Ankita and his colleagues constructed a polyurethane modified with antioxidant gallic acid (PUGA) and decellularized extracellular matrix dECM combined scaffold patch to deliver ASC-derived exosomes (67). Their results showed decreased fibrosis, promoted angiogenesis, reduced oxidative stress after application of patch, as well as improved cardiac function. Nowadays, due to their low immunogenicity, minimal tumorigenicity, and easy storage and transportation, exosomes have garnered increasing attention as a potential therapeutic tool for IHD.

## Does the tissue type or origin of ASCs affect their biological function?

Mammalian adipose tissue comes in three types: white, brown, and beige (68). White adipose tissue (WAT), as the primary energy-storing organ and chief culprit of obesity, occurs in various locations, including intraabdominal and subcutaneous sites; brown adipose tissue (BAT) exists in cervical, axillary, periadrenal, and perirenal area in the fetus and newborn and then transforms into WAT with aging. Nowadays, emerging shreds of evidences have supported the presence of BAT in adults human, located in the supraclavicular, perirenal, and deep neck region, with a thermogenic function (69). Beige adipose tissue, also known as brite adipose tissue, is a phenotype that arises from the “browning” of WAT upon cold exposure (70). Currently, there is limited research on the differences between ASCs obtained from BAT and WAT, partly due to scarcity of BAT depots, especially in humans. However, studies have shown enhanced proliferation, differentiation, and paracrine potential of ASCs isolated from BAT in contrast to WAT. For instance, ASCs obtained from pericardial and thymic depots exhibited longer doubling time compared to those from the subcutaneous or intraperitoneal region (71). Moreover, ASCs obtained from BAT have demonstrated spontaneous differentiation into cardiomyocytes, which can be further accelerated by chitosan hydrogel (25).

Except for tissue type, the organ origin of adipose tissue also affects the characteristics of ASCs. ASCs derived from the epicardial fat of cardiac patients have been found to induce a superior angiogenic effect and produce higher amounts of angiogenic, trophic, and inflammatory cytokines compared with ASCs from subcutaneous fat, which can result in worse heart function after MI due to their proinflammatory properties (36). Furthermore, scientists have demonstrated that subcutaneous ASCs and ASCs from the intraabdominal region, both of which belong to WAT, develop different proliferation and adipogenic differentiation potential. ASCs from subcutaneous adipose tissue display an enhanced adipogenesis potential, while progenitor cells isolated from the infrapatellar fat pad express higher levels of chondrogenic markers (72). Therefore, considering the distinct biological behavior of ASCS from different origins, it is crucial to select suitable cell candidates for preclinical application.

## Factors affecting the function of ASCs: aging, diabetes mellitus, and atherosclerosis

**Figure 2:**
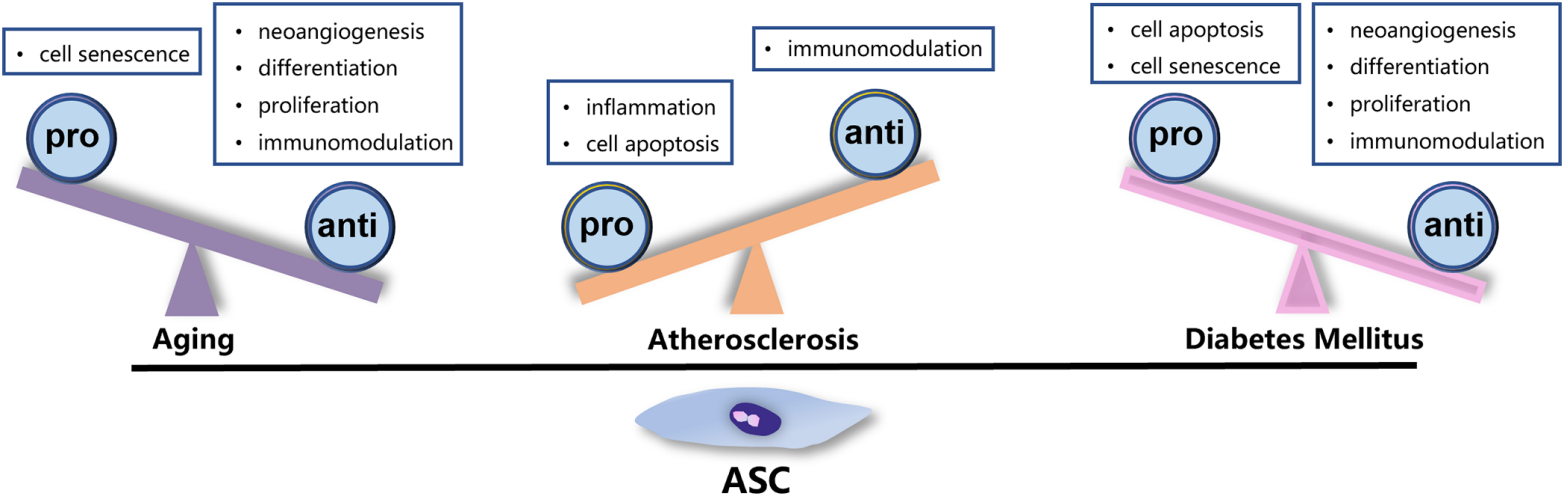
Factors affecting the function of ASCs. Aging, ATH, and DM exert negative impact on the characteristic and function of ASCs through different mechanisms.

Currently, autologous transplantation remains the predominant strategy for stem cell-based therapy. However, the viability and quality of stem cells can be significantly affected by the general health of donors. In clinical settings, patients undergoing cell transplantation usually suffer from systemic pathologies such as hypertension, diabetes, or autoimmune disease. The heterogeneity of ASCs remarkably imposes restrictions on the efficacy of autologous transplantation, highlighting the importance of selecting the optimal donor for allotransplantation. Given that patients with IHD are typically elderly and often have diabetes or atherosclerosis, these three factors have been studied extensively as a matter of course. The factors that impact the function of ASCs are detailed below and summarized in the accompanying figures (Figure 2).

## Aging

While the decrease in the number of ASCs with increasing age remains controversial (73), there is no denying that aging exerts a negative impact on the biological feature of ASCs. Firstly, studies have shown that the proliferative rate of ASCs obtained from older animals or individuals declined dramatically (74, 75). Additionally, ASCs derived from older donors exhibited typical senescence phenotypes, including an increased percentage of G1/G0 phase-arrested cells, decreased telomere length, elevated β-galactosidase activity, and binucleation as well (76). Moreover, the differentiation capability of ASCs from older donors was reduced, although it might be rescued by different cell culture procedures (77). Furthermore, the decline in function of ASCs from older patients was evident in their reduced production of pro-angiogenic factors such as VEGF, HGF, PIGF, and ANG, as well as their inferior attenuation of CD4^+^ T cells proliferation, indicating an age-associated reduction in paracrine and immunomodulatory capacity (78). Given that MSCs, including ASCs, play a crucial role in repair after injury, the decreased function due to aging can be a significant pathogenic factor in age-related pathologies such as atherosclerosis, diabetes, and arterial hypertension.

## Diabetes mellitus

In the microenvironment of patients with diabetes mellitus (DM), some subtle changes occurs, including hyperglycemia, excessive oxidative stress, mitochondrial dysfunction, proinflammatory cell status, and hypoxia, which inevitably impact ASCs to some extent (79, 80). Scientists have reported decreased proliferation, increased senescence and apoptosis, and downregulated VEGF expression in diabetic ASCs (81, 82), as well as impaired EC differentiation manifested by declined vWF and CD31 compared with healthy counterparts (83, 84). In addition to VEGF, diabetic ASCs secreted lower levels of angiogenic factors than healthy ASCs, including FGF, PDGF, SDF-1, osteopontin, insulin-like growth factor binding protein-3, and monocyte chemoattractant protein-1 (82). They failed to form tubular structures in Matrigel, and their potential of promoting angiogenesis was notably impaired (85). Consistent with *in vitro* study results, the degree of vascularization and wound healing in the mice wound model with implantation of diabetic ASCs was significantly reduced compared with that with nondiabetic ASCs (82, 86). Besides, the adipogenesis (84) and osteogenic differentiation (87) potential of diabetic ASCs also declined, indicating a loss of cell stemness in the hyperglycemic environment. It has been reported that hyperglycemia generates advanced glycation end products (AGEs), which induces osteoclast formation and apoptosis of osteoblasts (87). However, this effect may differ in humans, as ASCs from DM patients demonstrated robust osteoblast differentiation, indicating the complexity of ASCs biological behavior (88). Moreover, ASCs derived from DM patients exhibit an inflammatory phenotype, characterized by activation of NLRP3 inflammasome and subsequent alterations in immunomodulatory capacity (89). Furthermore, in pressure-ulcer model, mice treated with nondiabetic ASCs displayed less infiltration of inflammatory cells into the dermis and more new blood vessels *in situ* during the first 2 weeks compared with diabetic ASCs; however, these advantages disappeared afterward (43). The underlying mechanism of adverse effect of hyperglycemic microenvironment of DM on ASCs remains to be further explored.

## Atherosclerosis

ASCs play a protective role in the development of ATH. In turn, ASCs from ATH subjects (ATH-ASCs) exhibit distinct features compared with those isolated from non-ATH donors. Scientists have found elevated intracellular reactive oxidative stress (ROS) and mitochondrial ROS in ATH-ASCs (90). ROS activates NF-κB as a secondary messenger, leading to the increased accumulation of HIF-1α and upregulated expression of pro-inflammatory cytokine and chemokine (91). The ROS scavenger N-acetyl-L-cysteine can reduce the secretion of these cytokines in ATH-ASCs, enhance their survival and immune potency. Additionally, patients with ATH displayed a higher level of CD4+ T cells activation compared to those without ATH, and ASCs from non-ATH patients demonstrated superior inhibition on proliferating CD4^+^ T cells (78). Therefore, it is evident that ATH significantly impairs the immunomodulatory function of ASCs and negatively affect their therapeutic efficiency. In this case, it is advisable to exclude ATH subjects from the selection of suitable donors for regenerative medicine.

## Engineered ASCs

The primary obstacles to effective cell therapy include inadequate cell retention and low survival rates, which limit further clinical application. For decades, extensive studies have focused on encapsulation of ASCs within a variety of biomaterials to enhance their delivery and retention in ischemic myocardium. For instance, Follin, et al. (92) embedded human ASCs in an alginate hydrogel and reported no adverse effects on cell viability, phenotype, immunogenic properties, or paracrine activity. Similarly, the fullerenol/alginate hydrogel was found to dramatically scavenge the superoxide anions in ischemic area, thereby enhancing ASCs retention and survival, and ultimately promoting cardiac recovery in a rat MI model (93). Besides, chitosan hydrogel has been considered an ideal carrier due to its components facilitating cardiac differentiation of ASCs (25). *In vivo* studies have revealed enhanced survival of engrafted ASCs, increased generation of ASCs-derived cardiomyocytes, improved angiogenesis, and preserved cardiac healing. In addition to various hydrogels (94, 95), a growing number of biomaterials have also shown promise in enhancing cell retention and promoting cardiac repair, including Matrigel (96), collagen type-1 scaffold (97), superparamagnetic iron oxide nanoparticles (98), poly(lactic-co-glycolic acid) (PLGA) (99), decellularized pericardium (100), poly(ɛ-caprolactone-co-glycolic acid) and poly(ethylene glycol) (tri-PCG) (101), conductive electrospun nanofibers (102), injectable cryogels (103), et al. Biomaterials for ASCs delivery have demonstrated great potential in preclinical studies and provide guidance for their application in the treatment of MI in human clinical studies.

## Clinical trials

Based on various *in vitro* and *in vivo* studies, ASCs transplantation has been widely utilized for IHD treatment in clinical trials. Quantities of clinical trials have been conducted to assess the safety, feasibility, and effectiveness of ASC in individuals with heart diseases (Table 2). However, due to the complexity and severity of IHD, the current clinical applications of ASCs in the field remain in the early phases (phage I/II).

**Table 2:** Completed and ongoing trials of ASCs in heart disease.

Clinicaltrials.gov identifier	Study design	Disease type	Route of delivery	Endpoint	Enrolled number	Status
NCT01709279	Single group assignment, open label	Ischemic heart failure	Intracoronary injection	All cause harmful events	6	Enrolling by invitation
NCT00426868	Randomized, parallel assignment, phase I	Ischemic heart disease	Transendocardial injections	Safety, feasibility	27	Completed
NCT00442806	Randomized, parallel assignment, phase I	Acute myocardial infarction	Intracoronary injection	Safety, feasibility	14	Completed
NCT01449032	Double-blind, parallel assignment, phase II	Chronic ischemic heart disease	Intramyocardial injection	Exercise test, clinical evaluation	60	Completed
NCT03746938	Single group assignment, open label, phase I	Heart failure with reduced ejection fraction	Collagen membrane seeded	MACCE	10	Recruiting
NCT02387723	Single group assignment, open label, phase I	Heart failure	Intramyocardial injection	Safety, cardiac efficacy	10	Completed
NCT02673164	Double-blind, parallel assignment, phase II	Heart failure	Intramyocardial injection	LVESV, safety	133	Active, not recruiting
NCT03092284	Double-blind, parallel assignment, phase II	Heart failure	Intramyocardial injection	LVESV, safety	81	Active, not recruiting
NCT01556022	Randomized, parallel assignment, phase II	Myocardial ischemia	Intramyocardial injection	Safety, cardiac function	28	Completed
NCT01216995	Randomized, parallel assignment, phase II	Acute myocardial infarction	Intracoronary injection	Reduction in infarct size, MACCE	23	Completed
NCT02052427	Randomized, parallel assignment, phase II	Myocardial ischemia	Intracoronary injection	Cardiac function, safety	3	Completed
NCT04005989	Double-blind, parallel assignment, Phase III	Ischemic heart disease	Intracoronary injection	All cause harmful events	40	Not yet recruiting
NCT03797092	Randomized, parallel assignment, phase II	Non-ischemic dilated cardiomyopathy	Intramyocardial injection	LVESV, LVEF	30	Recruiting
NCT02673164	Randomized, parallel assignment, double-blind, placebo-controlled, phase II	Heart failure with reduced ejection fraction	Intramyocardial injection	LVESV, safety	133	Completed

MACCE, major adverse cardiac and cerebral events; LVESV, left ventricular end-systolic volume.

The Netherlands clinical trial conducted by Jaco H. Houtgraaf and his colleagues in 2007 (NCT00442806) (104) was the first clinical trial using ASCs in IHD. This trial was a randomized, parallel assigned, double-blinded clinical trial for ST-segment elevation acute myocardial infarction. Totally 14 patients were enrolled and after 6 months of follow-up, the study uncovered that intracoronary injection of freshly isolated ASCs was safe and effective, with no adverse effect related to ASCs implantation, no decline of coronary blood flow, improved cardiac function, and reduction of scar formation. Besides, a recent 3-year follow-up MyStromalCell Trial (NCT01449032) (105) performed by Abbas Ali Qayyum and colleagues published the data of autologous ASCs treatment in 60 patients with chronic refractory angina. This study observed a marked decline in chest discomfort and a decrease in frequency of angina attacks in the ASCs group. However, no significant differences were observed between the two groups in the exercise tolerance testing. In their latest SCIENCE trial (NCT02673164) (106), allogeneic ASCs from healthy donors were injected intramyocardially into 133 IHD patients with reduced ejection fraction. Three-year follow-up data disclosed the safety of allogeneic ASCs therapy. However, there were no significant differences in cardiac function including LVEF, LVESV, and LVEDV between ASCs and placebo group. In clinical trial NCT03797092 (107), scientists utilized the cryopreserved product ASCs from healthy donors to treat patients with IHD and observed improved cardiac function without ASCs-related immune response after 6-months of follow-up. In another research followed-up for 12-month, authors utilized cardiac magnetic resonance to evaluate the improvement of cardiac function. Thirteen patients with IHD were enrolled and accepted ASCs implantation. Their results disclosed increased stroke volume and left ventricle ejection fraction, as well as improved cardiac output after 12 months of follow-up (108).

The completed clinical trials in Table 2 showed the safety and efficacy of ASCs therapy in IHD without exception. However, most studies evaluated the major adverse cardiac and cerebral events (MACCE) as the adverse effect and cardiac function at a very early stage, which might overlook the potential risks and overstate the benefit of ASCs implantation. Inflammation and embolism have been recognized as the major blight of ASCs application. Longtime follow-up clinical trials with a large sample size will provide more supportive data for this procedure.

## Conclusion

ASCs are readily obtained, with minimal invasiveness, high yield, low immunogenicity, and no ethical issues, which enable them an innovative option in regenerative medicine. Meanwhile, ASCs have exhibited great advantages both in autologous and allogeneic transplantation, especially in the commercialization of stem cell-based therapy. ASCs play a protective role in IHD through differentiation into cardiomyocytes and endothelial cells, but more importantly acting as “paracrine factories” where a large quantity of cytokines is produced to trigger angiogenesis and modulate the immune system. However, the heterogeneity of ASCs should attract more attention due to that tissue origin or health state of donors affects cell properties and functions. Therefore, a critical and proper criterion will enable us to select the appropriate donors for clinical applications. Moreover, to overcome the limitation of low retention after engraftment, engineered ASCs through biomaterials have exhibited great potential in preclinical studies. Continuously optimized delivery strategies are of great value in clinical transformation of ASCs.
